# Profiles of Human Papillomavirus Detection of the Multinucleated Cells in Cervical Smears

**DOI:** 10.3390/microorganisms9081575

**Published:** 2021-07-23

**Authors:** Kaori Okayama, Toshiyuki Sasagawa, Koji Teruya, Mizue Oda, Masahiko Fujii, Hirokazu Kimura, Mitsuaki Okodo

**Affiliations:** 1 Department of Health Science, Gunma Paz University Graduate School of Health Sciences, 1-7-1 Tonyamachi, Takasaki-shi, Gunma 370-0006, Japan; okayaman0811@std.kyorin-u.ac.jp (K.O.); h-kimura@paz.ac.jp (H.K.); 2 Department of Obstetrics and Gynecology, Kanazawa Medical University, 1-1 Uchinadadaigaku, Kahoku-gun, Ishikawa 920-0293, Japan; tsasa@kanazawa-med.ac.jp; 3 Department of Health and Welfare, Faculty of Health Sciences, Kyorin University, 5-4-1 Shimorenjaku, Mitaka-shi, Tokyo 181-8621, Japan; teruya@ks.kyorin-u.ac.jp; 4 Genki Plaza Medical Center for Health Care, 3-6-5 Iidabashi, Chiyoda-ku, Tokyo 102-0072, Japan; m-oda@genkiplaza.or.jp; 5 Department of Medical Technology, Faculty of Health Sciences, Kyorin University, 5-4-1 Shimorenjaku, Mitaka-shi, Tokyo 181-8621, Japan; fujiim1951-1011@tbz.t-com.ne.jp

**Keywords:** human papillomavirus, multinucleated cell, koilocyte, Papanicolaou test, microdissection, cervical intraepithelial neoplasia

## Abstract

Many genotypes of human papillomaviruses (HPVs) may lead to morphological changes in cells, resulting in various atypical cells, such as multinucleated cells (MNCs) and koilocytes, in the cervix. However, the relationships between the profiles of HPV genotypes and MNCs are not exactly known. Thus, this study comprehensively profiles the HPV genotypes in MNCs using a microdissection method. HPV genotypes and MNCs were detected in 651 cases with an abnormal Pap smear by liquid-based cytology. Specific HPV genotypes were also detected, including HPV16, 34, and 56, which might be associated with MNCs. This result suggests that the high-risk HPV genotypes, such as HPV16 and 56, are associated with the atypical changes in MNC morphology from normal cervical cells. The results also show that MNCs may be a predictor of squamous intraepithelial lesion.

## 1. Introduction

Over 100 genotypes of human papillomavirus (HPV) have been confirmed to be associated with tumorigenic diseases, including benign (warts) and malignant lesions (carcinomas) [1,2]. Some of them are cancer-associated genotypes (high-risk types), including HPV genotype 16 (HPV16), HPV18, and HPV52 [3,4]. Previous studies have also suggested that these high-risk genotypes are the leading causes of cervical cancers [5,6]. Various atypical cells, including koilocytes and multinucleated cells (MNCs), have also been confirmed in cervical smears [7]. The change from a normal cell to an atypical cell may be due to HPV infection [7]. Our previous study suggested that many HPV genotypes were detected in koilocytes, and their morphological changes may be due to these HPV infections [8]. MNC is reportedly a predictor of HPV-infected cells [9]. Moreover, it may be associated with transformation to cancer cells [10,11,12]. However, the relationships between the profiles of the HPV genotypes and MNCs are not well-understood.

The screening tools for cervical cancer are the Papanicolaou (Pap) and HPV tests. These tests permit a detailed observation of the cytopathology of cervical smears [13,14]. In general, cytopathological malignancy classifications (four grades) may be performed using the Bethesda system [15,16]. These classifications are useful for the prediction of cervical cancer [15,16]. In this study, the relationships between the profiles of the HPV genotypes, cytopathological malignancy, and MNCs in Pap smears were studied using a microdissection (MD) method.

## 2. Materials and Methods

### 2.1. Clinical Samples

A total of 1053 samples with cervical intraepithelial neoplasia (CIN) were obtained from patients during follow-ups at the Genki Plaza Medical Center for Health Care, Tokyo, Japan between 2014 and 2018. The mean patient age was 38 years (range, 20–67 years). The data on HPV vaccination history were not collected. Of the 1053 samples, 651 SurePath™ (Becton, Dickinson and Company, Franklin Lakes, NJ, USA) liquid-based cytology (LBC) samples had atypical squamous cells of undetermined significance (ASC-US) + based on the Bethesda system [15]. The 651 Pap smears had the following diagnoses: ASC-US (137 samples), low-grade squamous intraepithelial lesion (LSIL; 202 samples), atypical squamous cells and high-grade squamous intraepithelial lesion could not be ruled out (ASC-H; 54 samples), and high-grade squamous intraepithelial lesion (HSIL; 258 samples). Squamous cell carcinoma was detected in two of the 1053 samples, but these were excluded due to their small number. Two cytotechnologists strictly evaluated all the Pap smears for the presence or absence of MNCs in a blinded manner. For the diagnosis, the cytotechnologists resolved any discrepancies by viewing the cells simultaneously. The association between the koilocytes and HPV genotypes was also determined [8].

### 2.2. Ethical Approval

After obtaining written informed consent from the subjects, the samples were collected. The Ethics Committee on Human Research of the Kyorin University (2019-10) and Gunma Paz University (PAZ18-37) approved the study protocol, and it was implemented in accordance with the approved guidelines.

### 2.3. Human Papillomavirus Genotyping on the Whole LBC Samples

HPV genotyping was performed on the whole LBC samples to assess the association between the cytological signs on Pap smears and HPV genotypes. DNA from the residual liquid of the whole LBC samples were collected using the hot sodium hydroxide method [17]. Cell pellets were lysed with 50 μL of alkaline lysis solution (25 mM NaOH and 0.2 mM ethylenediaminetetraacetic acid (EDTA); pH, 12.0) for 30 min at 95 °C. Then, the lysed cells were neutralized with 0.04 M Tris-HCl (pH 5.0), centrifuged at 13,200 rpm for 1 min, and directly used as the DNA template. Using polymerase chain reaction (PCR), the presence of human β-actin in cells was determined, which served as the internal standard for genotyping. All HPV genotypes tested positive for human β-actin, demonstrating that an amplifiable quality of DNA was extracted from the specimens. The HPV-typing assay method utilized a uniplex E6/E7 PCR, as previously described by Okodo et al. [18]. This method can detect 39 mucosal HPV types, including 12 high-risk types (HPV16, 18, 31, 33, 35, 39, 45, 51, 52, 56, 58, and 59) and other genotypes (HPV 6, 11, 26, 30, 34, 40, 42, 44, 53, 54, 55, 61, 62, 66, 67, 68, 69, 70, 71, 73, 74, 81, 82, 84, 85, 89, and 90) [19] from as few as 100 viral copies, with no cross-reactivity across all the HPV genotypes. This PCR method may occasionally provide false-positive results; to eliminate the possibility of DNA contamination, each round of PCR was performed with negative controls using DNase-free water. A plasmid DNA containing the whole HPV genome was used as the positive control.

### 2.4. HPV Genotyping of Manually Microdissected Multinucleated Cells

MD was used to detect HPVs directly from MNCs on Pap smear slides [8,20]. The cell pellets were taken and analyzed from 11 randomly selected LBC samples with MNCs infected with multiple HPV genotypes, including the high-risk genotypes that were less likely to induce multinucleation based on logistic regression.

Each cell pellet was mounted in a single thin layer on a microscope slide and fixed with 95% ethanol. Two cytotechnologists reviewed the Pap smear slides, and certain MNCs were selected for sampling. The selected cells were individually photographed; then, the slides were soaked in xylene to remove the cover slip. Then, the xylene was washed away with 100% ethanol. Subsequently, under a stereomicroscope, the chosen cells were collected by gently picking up the edge of the cell with the point of a 27-G injection needle. The cells attached to the point of the needle were precisely transferred to each tube containing the alkaline lysis solution. DNA isolation and Uniplex E6/E7 PCR were performed for the HPV genotyping of the MD samples, as previously described.

### 2.5. Statistical Analysis

SPSS version 25.0 (SPSS Inc., Chicago, IL, USA) was used for the statistical analyses. The differences between the groups were examined using residual analysis. In addition, a stepwise logistic regression with backward elimination and a likelihood ratio test for model building were used to evaluate the HPV genotypes associated with MNCs. The goodness-of-fit was assessed using the Hosmer–Lemeshow test. All of the HPV genotypes were entered into the model; then, the nonsignificant genotypes were removed from the model one at a time. Moreover, the odds ratio (OR), confidence intervals (CIs), and *p*-values were estimated in each step. In all cases, a *p*-value of less than 0.05 was considered statistically significant.

## 3. Results

### 3.1. Relationships among the Multinucleated Cells, Cytologic Classification, and HPV Genotypes

The relationships among the MNCs, cytologic classifications, and HPV genotypes are summarized in Table 1, Table 2 and Table 3, respectively. MNCs were present in 19.7% (27/137), 50.5% (102/202), 14.8% (8/54), and 38.0% (98/258) of the ASC-US, LSIL, ASC-H, and HSIL cases, respectively (Table 1). MNCs were most frequently found in Pap smears with LSIL (approximately 50%). MNCs were also significantly associated with LSIL (*p* < 0.01). We estimated the correlation of the positive rate for MNCs with high-risk (Table 2) and other HPV genotypes, as determined by whole LBC PCR. Among cases with HPV infection, the high-risk genotypes with a high positive rate for MNCs included HPV16 and HPV56 (*p* < 0.01). As shown in Table 3, the positive rate for MNCs in the other genotypes was 66.7% (14/21) in HPV34-positive cases and 66.7% (10/15) in HPV67-positive cases, which were significantly higher (*p* < 0.01 and *p* < 0.05, respectively). The positive rate for MNCs were 60.0% (3/5) and 50.0% (3/6) in HPV35- and HPV45-positive cases, respectively. However, no significant relationships among HPV35, HPV45, and MNCs were found.

Subsequently, a binomial logistic regression analysis was used to determine the relationships between the detected HPV genotypes and atypical cells. The model was fit (Hosmer–Lemeshow test; *p* > 0.05). MNCs were significantly associated with the presence of HPV16 (*p* = 0.000; OR, 2.34; 95% CI, 1.58–3.48), HPV39 (*p* = 0.033; OR, 1.76; 95% CI, 1.05–2.95), HPV56 (*p* = 0.000; OR, 3.11; 95% CI, 1.89–5.12), HPV34 (*p* = 0.017; OR, 3.40; 95% CI, 1.24–9.32), and HPV67 (*p* = 0.043; OR, 3.43; 95% CI, 1.04–11.30).

### 3.2. HPV Genotype Detection by MD-PCR in Multinucleated Cells for Multiple Infection

**Figure 1:**
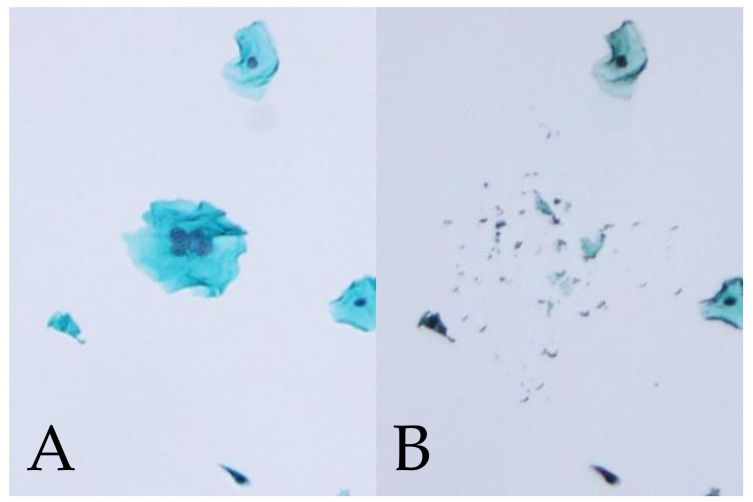
Excision of MNCs on Pap smear slides using the microdissection-based technique. (**A**): MNCs (Pap stain, ×100). (**B**): Appearance after the manual microdissection of one MNC with the point of a 27-G needle under a microscope.

Based on the correlation between the MNCs and HPV genotypes, MD-PCR was performed on the genotypes that showed *p* < 0.01. MD was performed in cases with MNC (Figure 1). Table 4 compares the HPV genotypes in MNCs based on MD-PCR. The whole LBC PCR results from the 11 cases of infection showed that multiple genotypes, including HPV16, 56, and 34, were likely to induce multinucleation. From the 11 cases, 54 MD samples of MNCs were taken. Of these, 25 (46.3%) were HPV-positive, with either HPV16, 56, or 34 genotypes, while 29 MD samples were HPV-negative. Therefore, multinucleation might be associated with specific HPV genotypes.

## 4. Discussion

We studied the relationships between MNC, cytopathological classification by Bethesda system, and HPV infection profiles in cervical smears stained using the Pap and microdissection methods. The main highlights of this study are as follows: First, MNCs were most frequently found in LSIL (Table 1). Second, various low-risk and high-risk HPV genotypes were detected in the whole cells, while common genotypes, such as HPV16 and 56, were detected in MNCs (Table 4). These results suggest that the high-risk HPV genotypes, such as HPV16 and 56, are associated with the atypical change of normal cells into MNCs. Thus, such cells may be a candidate for squamous intraepithelial lesion cells. To the best of our knowledge, this finding may be the first of its kind.

In general, the malignancy grade of cervical cells may be evaluated by nuclear atypia and nuclear/cytoplasm rates [15]. Based on these microscopic findings, previous studies further evaluated various atypical cell types, including MNCs, koilocytes, binucleated cells, giant cells, parakeratotic cells, and smudge cells, following the Bethesda system [7,15]. These morphological changes may suggest HPV infection. However, it is not known which cells will progress to cancer in the future, while those with the high-risk HPV genotypes may commonly become cervical cancer cells. This study also found that MNCs are a candidate for squamous intraepithelial lesion cells, because various high-risk genotypes, including HPV16 and 56, infected the cells. At present, HPV34 is not classified as a high-risk genotype, while other studies suggested that this genotype, in addition to HPV16 and 56, is also associated with cervical cancer [21]. Taken together, MNCs could be predictors of both high-risk HPV genotype infections and cervical cancer cells.

HPV profiling was also performed in the koilocytes in the cervical smears using MD [8]. Various HPV genotypes, including HPV39, 53, 56, and 66, were detected, while HPV16 was not detected in the samples. Such differential profiles of HPV genotype infections between the MNCs and koilocytes may reflect the morphological changes in the cervical epithelium.

In general, MNCs are found in other infections due to Chlamydia or human herpes virus type 2 [22,23,24]. These cells may not progress to cancer cells in the future [22]. Chlamydia- or herpes virus-infected cells have characteristic findings, such as cytoplasmic inclusion bodies or nuclear inclusion bodies, respectively. These can be distinguished from HPV-infected MNCs. Thus, to differentiate their effects from the cytological signs of HPV infection, laboratory tests for the pathogen profiles should be performed.

There are some limitations in this study. First, using the MD method it is possible that there was a contamination of HPV genotypes between the MNCs and other cells in the smears because the PCR method used in this study was specific and highly sensitive. Meanwhile, the positive rate of MD-PCR for MNCs was not very high because the target cell was a single cell, and the viral load might have been below the detection limit. Another possible explanation is the influence of PCR inhibitors on microscope slides. Thus, it may be necessary to consider microscope slides that are suitable for MD-PCR. It was also not demonstrated whether specific HPV genotypes were detected from microdissected MNCs on the histological specimens. Thus, it is unclear whether MNCs were detected in CIN tissues, even if they were significantly associated with LSIL. However, the contamination of adjacent cells is inevitable when single cells are isolated using LCM [25]. Despite these limitations, using MD and HPV genotyping with a sensitive PCR, it was demonstrated that koilocytes are a cytological sign of particular HPV genotypes [8]. In this study, it was not possible to mention the mechanism of how HPV16 and 56 formed MNCs. However, we believe that this analysis of the relationships between the high-risk HPV genotypes and MNCs in Pap smears are significant.

In conclusion, the present study showed that MNCs may be a predictor of cervical cancer cells. However, other atypical cells, such as binucleated cells, parakeratotic cells, giant cells, and smudge cells, were also found in the cervical smears. To predict the progression of candidates into cervical cancer cells, further studies may be needed to assess the profiles of HPV infection in these cells.

## Figures and Tables

**Table 1:** Correlation between the multinucleated cells (MNCs) in cervical smears and cytologic classifications in 651 samples.

MNC	ASC-US	LSIL	ASC-H	HSIL
	*n* = 137	*n* = 202	*n* = 54	*n* = 258
Present	19.7%	50.5%	14.8%	38.0%
	(27/137)	(102/202)	(8/54)	(98/258)
Absent	80.3%	49.5%	85.2%	62.0%
	(110/137)	(100/202)	(46/54)	(160/258)

HPV, human papillomavirus; MNC, multinucleated cell; ASC-US, atypical squamous cells of undetermined significance; LSIL, low-grade squamous intraepithelial lesion; HSIL, high-grade squamous intraepithelial lesion

**Table 2:** Correlation between the MNCs in cervical smears and high-risk human papillomavirus (HPV) genotypes.

MNC	High-Risk HPV Genotypes											
	16	18	31	33	35	39	45	51	52	56	58	59
	*n* = 143	*n* = 35	*n* = 53	*n* = 23	*n* = 5	*n* = 71	*n* = 6	*n* = 72	*n* = 151	*n* = 81	*n* = 143	*n* = 9
Present	49.0%	34.3%	43.4%	21.7%	60.0%	45.1%	50.0%	38.9%	29.1%	55.6%	28.0%	44.4%
	(70/143)	(12/35)	(23/53)	(5/23)	(3/5)	(32/71)	(3/6)	(28/72)	(44/151)	(45/81)	(40/143)	(4/9)
Absent	51.0%	65.7%	56.6%	78.3%	40.0%	54.9%	50.0%	61.1%	70.9%	44.4%	72.0%	55.6%
	(73/143)	(23/35)	(30/53)	(18/23)	(2/5)	(39/71)	(3/6)	(44/72)	(107/151)	(36/81)	(103/143)	(5/9)

HPV, human papillomavirus; MNC, multinucleated cell.

**Table 3:** Correlation between the MNCs in cervical smears and other HPV genotypes.

MNC	Other HPV Genotypes													
	6	11	26	30	34	40	42	44	53	54	55	61	62	
	*n* = 22	*n* = 4	*n* = 0	*n* = 14	*n* = 21	*n* = 14	*n* = 58	*n* = 10	*n* = 59	*n* = 21	*n* = 25	*n* = 30	*n* = 44	
Present	50.0%	50.0%	–	28.6%	66.7%	21.4%	24.1%	20.0%	45.8%	19.0%	28.0%	26.7%	34.1%	
	(11/22)	(2/4)		(4/14)	(14/21)	(3/14)	(14/58)	(2/10)	(27/59)	(4/21)	(7/25)	(8/30)	(15/44)	
Absent	50.0%	50.0%	–	71.4%	33.3%	78.6%	75.9%	80.0%	54.2%	81.0%	72.0%	73.3%	65.9%	
	(11/22)	(2/4)		(10/14)	(7/21)	(11/14)	(44/58)	(8/10)	(32/59)	(17/21)	(18/25)	(22/30)	(29/44)	
**MNC**	**Other HPV Genotypes**													
	**66**	**67**	**68**	**69**	**70**	**71**	**73**	**74**	**81**	**82**	**84**	**85**	**89**	**90**
	***n* = 22**	***n* = 15**	***n* = 33**	***n* = 2**	***n* = 10**	***n* = 24**	***n* = 9**	***n* = 51**	***n* = 24**	***n* = 39**	***n* = 17**	***n* = 0**	***n* = 17**	***n* = 37**
Present	40.9%	66.7%	18.2%	0.0%	50.0%	50.0%	55.6%	39.2%	29.2%	30.8%	23.5%	–	47.1%	43.2%
	(9/22)	(10/15)	(6/33)	(0/2)	(5/10)	(12/24)	(5/9)	(20/51)	(7/24)	(12/39)	(4/17)		(8/17)	(16/37)
Absent	59.1%	33.3%	81.8%	100.0%	50.0%	50.0%	44.4%	60.8%	70.8%	69.2%	76.5%	–	52.9%	56.8%
	(13/22)	(5/15)	(27/33)	(2/2)	(5/10)	(12/24)	(4/9)	(31/51)	(17/24)	(27/39)	(13/17)		(9/17)	(21/37)

HPV, human papillomavirus; MNC, multinucleated cell.

**Table 4:** Detection of MNCs by MD-PCR in multiple human papillomavirus (HPV)-infected samples.

Case No.	Cytologic Classification	HPV Genotype	
		WL-PCR	MD-PCR (MD-PCR Positive/MD Samples)
1	HSIL	16, 82	– (0/1)
2	HSIL	16, 58, 30	– (0/3)
3	LSIL	16, 34	16 (6/8), 34 (1/8)
4	LSIL	34, 6, 42, 61, 62, 67, 74	34 (3/3)
5	LSIL	56, 6, 90	56 (3/6)
6	ASC-US	56, 39, 73	56 (3/6)
7	HSIL	56, 51, 30, 34, 53, 67	56 (2/6)
8	LSIL	56, 6	56 (3/8)
9	LSIL	56, 55, 74	56 (4/9)
10	LSIL	56, 90	– (0/3)
11	LSIL	56, 55, 74	– (0/1)

The numbers in parentheses in the MD method indicate the ratio of the number of MD samples to the number of positive MD-PCR results in each case. HPV, human papillomavirus; ASC-US, atypical squamous cells of undetermined significance; LSIL, low-grade squamous intraepithelial lesion; HSIL, high-grade squamous intraepithelial lesion; MD, microdissection; PCR, polymerase chain reaction; WL, whole liquid-based cytology.

## Data Availability

The data presented in this study are available on request from the corresponding author. The data are not publicly available due to restrictions eg privacy or ethical.
